# 11q13 amplification status and human papillomavirus in relation to p16 expression defines two distinct etiologies of head and neck tumours

**DOI:** 10.1038/sj.bjc.6603394

**Published:** 2006-09-26

**Authors:** C C R Ragin, E Taioli, J L Weissfeld, J S White, K M Rossie, F Modugno, S M Gollin

**Affiliations:** 1 Department of Human Genetics, University of Pittsburgh Graduate School of Public Health, 130 DeSoto Street, Pittsburgh, PA 15261, USA; 2 Department of Epidemiology, University of Pittsburgh Graduate School of Public Health, Pittsburgh, PA 15261, USA; 3 The University of Pittsburgh Cancer Institute, Pittsburgh, PA 15232, USA; 4 Department of Oral Medicine and Pathology, School of Dental Medicine, Pittsburgh, PA 15232, USA

**Keywords:** HPV, 11q13, gene amplification, CDKN2A/p16, head and neck cancer, *TP53*

## Abstract

Two distinct etiologies of head and neck squamous cell carcinoma (HNSCC) have been proposed, DNA damage owing to tobacco and alcohol exposure and human papillomavirus (HPV) oncogene-mediated transformation. Common genetic alterations in HNSCC include *TP53* mutations, 11q13 amplification (amp) and *CDKN2A*/p16 mutations or promoter methlyation. However, in HPV+ HNSCC it is frequent to observe wild-type *TP53* and expression of p16. The relationship of this unusual pattern with 11q13 amp has not been tested. In a retrospective study on 125 HNSCC patients, only 17% (five out of 30) of HPV+ *vs* 44% (39 out of 89) of HPV − tumours expressed 11q13 amp (adjusted odds ratio (OR)=0.2, 95% confidence interval (CI)=0.1–0.6). A subpopulation of tumours (*n*=69) were classified according to the three molecular markers, *TP53*, p16 and 11q13 amp. In addition to wild-type *TP53*, and p16 expression, HPV+ tumours were more likely not to be amplified at 11q13 (OR=6.5, 95% CI=1.8–23.9). As HPV+ HNSCC lack the genetic alterations which are common in other tumours, we hypothesise that HPV infection may represent an early event in the HNSCC carcinogenic process, thus suggesting a distinct molecular pathway.

Although the majority of cases of head and neck squamous cell carcinoma (HNSCC) are attributed to tobacco and alcohol consumption (Blot *et al*, 1988; Merletti *et al*, 1989; Franceschi *et al*, 1990; Franceschi *et al*, 1999), morphologic signs of human papillomavirus (HPV) infection and detection of the viral antigens in oral squamous cell carcinomas were first reported in 1983 (Syrjanen *et al*, 1983). Increasing evidence from large case–control studies of HPV prevalence in head and neck tumours suggests that this virus may be an additional independent risk factor for a subset (∼25%) of HNSCC (Smith *et al*, 1998; Gillison *et al*, 2000; Herrero *et al*, 2003).

Head and neck squamous cell carcinoma frequently carries numerous genetic alterations, such as mutations, chromosomal losses, gains and amplifications (amp) (Gollin, 2001). Common genetic alterations in HNSCC involve *TP53*, *CDKN2A*/p16 and chromosomal band 11q13. Amplification of 11q13 is among the most common sites of gene amplification, observed in a number of cancers (Schraml *et al*, 1999), and in approximately 45% of HNSCC (Lese *et al*, 1995; Schuuring, 1995; Gollin, 2001). A number of putative oncogenes map to this region; among them, *CCND1* (cyclin D1) is thought to play a role in tumorigenesis. Cyclin D1 is an important cell cycle protein that binds cyclin-dependent kinase (CDK) 4 or 6 to phosphorylate and inactivate the RB1 protein thus driving the cell from G1 into S-phase. Therefore, overexpression of cyclin D1 may lead to deregulate cell cycling from G1 to S-phase and may result in a selective growth advantage for the affected cell.

*CDKN2A*/p16 opposes cyclin D1 function by negatively regulating RB1 phosphorylartion and a genetic (loss of heterozygosity (LOH)) or epigenetic (promoter methylation) alteration in this gene is frequently observed, resulting in a lack of p16 expression in most HNSCC (Weber *et al*, 2003). *TP53*, also is a frequently mutated tumour-suppressor gene in a number of cancers, including about 45% of HNSCC (Sidransky, 1995).

In contrast to most HNSCC, HPV-positive tumours are more likely to have wild-type *TP53*. Human papillomavirus-infected cells accomplish G_1_ to S-phase transition through the targeted degradation of pRb by the viral E7 oncoprotein and consequently, *CDKN2A/*p16 is overexpressed. Correlations between genetic alterations at *TP53*, 11q13 and p16 expression with HPV have not been evaluated. We report a retrospective study examining the association of HPV with these molecular risk factors (*TP53* mutation, p16 protein expression and 11q13 amp) in patients with first primary head and neck tumours.

## MATERIALS AND METHODS

### Study population

The study population (*N*=185) included patients undergoing surgical resection of their tumours between September 1992 and February 1997, following informed consent under a protocol approved by the University of Pittsburgh Biomedical Institutional Review Board. Patients with recurrent tumours, second primaries and metastatic tumours (lymph node and distant) at the time of enrollment were excluded from this analysis (*n*=58). The remaining subjects (*n*=127), with first primary tumours were enrolled. Human papillomavirus could be tested in only 125 of 127 tumours; therefore, the number of patients included in this analysis was 125.

### Head and neck database

The head and neck database was established by CCRR, JSW and JLW in June 2004. Information, including demographics, smoking and alcohol use, family history, tumour site, clinical characteristics of the initial primary tumour and subsequent tumours, follow-up data (such as disease outcome and time to next disease occurrence) through May 2004 were obtained from the University of Pittsburgh Tumor Registry. Patients' clinical and demographic information from the Tumor Registry was re-verified from each patient's original deidentified and coded questionnaires, pathology and surgical reports. All data from the Tumor Registry was combined with the corresponding patient's laboratory data (i.e. *TP53* mutation, 11q13 amp, p16 expression and HPV status and genotype) to form the head and neck database. This database contains clinical, treatment and follow-up information for the first surgical resection at the time of enrollment and all subsequent resections for all patients enrolled in the study.

Smoking status was defined as follows: never smoker (never smoked), current smoker (recorded as an ever smoker who had not quit at the time of enrollment), former smoker (quit smoking at least 1 month before enrollment in the study). Alcohol use was defined as follows: never drinker (never consumed alcohol), current drinker (had not quit alcohol consumption at the time of enrollment) and former drinker (quit alcohol consumption before enrollment). For the duration of alcohol use, each patient was categorised as never (never consumed alcohol), short-term (consumed alcohol socially or for less than 10 years), or long-term (consumed alcohol for 10 years or more, irrespective of consumption habit) users. Tumour site, histology, stage and grade were classified according to the American Joint Committee on Cancer (AJCC) ICD9 codes, ICD-morphology, stage and grade classification, respectively. Sites of the oral cavity included cheek, retromolar area, alveolar ridge, oral tongue, palate, floor of mouth and overlapping lesions of other and unspecified parts of the mouth. The oropharynx included sites involving the base of tongue, soft palate, tonsil and overlapping lesions of the oral cavity and pharynx. Analyses of *TP53* mutation status (Law *et al*, 1995) and (Gollin *et al*, unpublished data), p16 immunohistochemistry (unpublished data) and 11q13 amp (Shuster *et al*, 2000; Lese *et al*, 1995) were performed in earlier unpublished studies unless otherwise noted. Briefly, *TP53* mutation status was determined by automated sequencing of exons 5–8, p16 immunohistochemistry was performed on paraffin sections of formaldehyde-fixed tissue using a monoclonal antibody to full length human p16 protein (Vector Labs, Burlingame, CA, USA). Cells were considered p16-positive if more than 5% of cells in a tumour island or mucosal surface stained visibly stronger than the negative control. When more than 5% of the cells were stained weakly, the result was considered to be weakly positive for p16. Amplification of 11q13 was assessed using fluorescence *in situ* hybridisation (FISH) probes for *FGF3/INT2, FGF4/HST1* or *CCND1* (Lese *et al*, 1995; Shuster *et al*, 2000).

### Human papilloma virus testing and genotyping

Extracted DNA was available from 125 tumours. Polymerase chain reaction (PCR) amplification of a *β*-globin gene fragment was performed using the PCO4 and GH20 primers (Saiki *et al*, 1986) to confirm that the extracted DNA was amplifiable. All samples were positive for the *β*-globin amplicon (*n*=125). Human papillomavirus status was determined by a nested real-time PCR assay using consensus HPV primers PGMY09/11/HMB01 (Gravitt *et al*, 2000) and GP5+/6+ primers (de Roda Husman *et al*, 1995). DNA amplification was performed using the LightCycler® FastStart DNA Master^PLUS^ SYBR Green I kit on a LightCycler® 2.0 system (Roche Diagnostics, Indianapolis, IN, USA). All DNA amplification reactions were set up according to the manufacturer's protocol (using 3 mM MgCl_2_ and 0.3 *μ*M each PGMY09/11/HMB01 primers (for the primary PCR reaction) or 1.0 *μ*M each GP5+/GP6+ primers (for the nested PCR reaction)) and were performed in a separate room from that used for the DNA amplification steps. For all HPV-positive samples from the previously described PCR protocol, confirmation of the appropriately sized amplicon was carried out by running an aliquot of each sample on a 10% PAGEr® polyacrylamide gel (Cambrex Bio Science, Rockland, ME, USA), at 250 volts for 55 min. The gel was stained with the highly sensitive GelStar® nucleic acid stain (Cambrex Bio Science, Rockland, ME, USA) and the 150 bp GP5+/6+ amplicon was visualised using a Clare Chemical Dark Reader transilluminator (Clare Chemical Research, Dolores, CO, USA).

HPV genotyping was performed using the Linear Array HPV Genotyping kit (Roche Diagnostics, Indianapolis, IN, USA). Briefly, the assay involved amplification of samples by PCR using a master mix which contained biotin-labelled primers for 33 HPV genotypes (6, 11, 16, 18, 26, 31, 33, 35, 39, 40, 42, 45, 51, 52, 53, 55, 56, 59, 61, 62, 64, 66, 68, 69, 70, 72, 73(MM9), 81, 82(MM4), 83(MM7), 84(MM8), IS39 and CP6108) as well as the human *β*-globin gene. The PCR products were chemically denatured and hybridised to linear array strips which contained specific and one cross-reactive oligonucleotide probes for the HPV genotypes listed above as well as a high and low concentration of a *β*-globin probe. The specific genotypes were visualised using streptavidin-horseradish peroxidase conjugate and a substrate solution containing hydrogen peroxide and 3,3′,5,5′-tetramethylbenzidine which yielded a blue precipitate at the probe positions where hybridisation occurred.

### Pyrosequencing

Ten samples, which were positive by nested-PCR, were not able to be genotyped using the Linear Array kit because they were below the detectable limit of the assay. Human papillomavirus genotyping of these samples was accomplished by pyrosequencing (Biotage, Foxboro, MA, USA) performed according to the manufacturer's instructions. Briefly, the nested PCR was performed as described above. The GP6+ primer was biotinylated at the 3′-end. The PCR product was denatured to obtain single stranded DNA, followed by pyrosequencing using the GP5+ as the sequencing primer. The following dispensation order was used GCACTACGCTAGCTACTACTGACTGACTGACTGACTGACTGACTGACTGACTGACTGACTGACTG. To identify the HPV genotype, sequencing results were aligned with the expected sequences for the 37 most common HPV genotypes or blasted against HPV sequences in the NCBI database.

### Statistical analyses

Clinical and laboratory risk factors for the study population were extracted from our head and neck database and imported to a statistical software package for analysis. Statistical analyses were performed using the Intercooled STATA (version 8.2) software (StataCorp. LP, College Station TX, USA). Factors associated with HPV status were selected based on cross-tabulations and univariate logistic regression analyses. Cross-tabulations were analysed using the *χ*^2^ test or Fisher's exact test, where appropriate. Logistic regression was used to perform the analyses of associations between demographic, clinical and pathologic variables with HPV status. Crude odds ratio (OR) were first calculated. Univariate analyses for the association of selected variables with 11q13 amp status were performed in the subset of HPV-negative subjects to identify possible confounding factors of association. Potential confounders were identified based on *P*-values ⩽0.1. Logistic regression was used to calculate the relative odds of HPV status with 11q13 amp after adjusting for the potential confounders (age, tumour stage and site).

## RESULTS

### Study population demographics

Table 1 summarises the patient demographics. Approximately 64% (80 out of 125) of the patients were males and 36% (45 out of 125) were females. The majority of patients were current smokers (*n*=76, 61%). The majority of the study population (65%) was dead by the last follow-up (May 2004). Our study population ranged in age from 18 to 92 years; the median age at diagnosis was 63 years, and the median age at death was 67 years.

### Human papillomavirus prevalence and genotype in tumour specimens

Twenty-four percent (30 out of 125) of the tumours were positive for HPV. All HPV-positive tumours contained HPV16 whereas two out of 30 (7%) were coinfected with other HPV types. One had an additional low-risk HPV type 11 and the other had an additional high-risk HPV type 33 and possibly 52 (a positive hybridisation signal for cross-reactive HPV33 probe on the HPV linear array genotyping strip could not rule out the presence of HPV52) (Table 1).

### Distribution of tumour human papillomavirus status according to patient characteristics

The majority of tumours occurred in the oral cavity (91 out of 125, 73%). Human papillomavirus-positive tumours occurred at various sites throughout the oral cavity and pharynx (data not shown). Approximately 20% (18 out of 91) of tumours from the oral cavity were found to be HPV-positive, whereas a slightly higher proportion of HPV-positive tumours were observed in the oropharynx (35% (12 out of 34). When stratified by smoking status, seven HPV-positive tumours from the oropharynx of current smokers occurred in the base of tongue (3), soft palate (2), tonsil (1) and an overlapping lesion of the oropharynx (1). In contrast, the five HPV-positive tumours from the oropharynx of nonsmokers and former smokers occurred in the tonsil (3) and base of tongue (2) only.

Analyses of factors associated with a positive HPV status are summarised in Table 2. Human papillomavirus-positive tumours were less likely to occur in older individuals (<55 years. 40% were HPV-positive *vs* ⩾55 years, 19% were HPV-positive: adjusted OR=0.3, 95% confidence interval (CI)=0.1–0.8), and were less likely to carry amplified genes in chromosomal band 11q13. After adjusting for age, tumour site and stage, 11q13 amp status was still statistically associated with HPV status, with only 17% (five out of 30) of HPV-positive tumours *vs* 44% (39 out of 89) of HPV-negative tumours having 11q13 amp (adjusted OR=0.2, 95% CI=0.1–0.6).

We evaluated the pattern of tobacco and alcohol risk behaviours by HPV status. Although the majority of patients from both the HPV-positive and negative groups were ever smokers and ever drinkers (77 and 80%, respectively), these patients were no more likely to be HPV-positive than those who never smoked or consumed alcohol (OR=0.8, 95% CI=0.1–7.8). However, no statistical interaction between alcohol and drinking habits with HPV-positivity was observed.

Individuals with HPV-positive tumours were less likely to have subsequent tumours, either recurrences, metastases or new primaries (adjusted OR=0.4, 95% CI=0.2–1.0). Subsequent tumours that developed in HPV-positive patients (33%, 10 out of 30) were confined to head and neck sites. In contrast, among patients with HPV-negative tumours, 57% (54 out of 95) developed recurrences, metastases or new primaries at head and neck sites, as well as other sites throughout the body.

A higher proportion of HPV-positive tumours occurred in the oropharynx. Forty percent (12 out of 30) of HPV-positive tumours compared to 23% (22 out of 95) of HPV-negative tumours occurred in the oropharynx (adjusted OR=2.4, 95% CI=1.0–5.8). A high tumour stage (stage III & IV: adjusted OR=1.4, 95% CI=0.6–3.5) and family history of cancer (adjusted OR=1.3, 95% CI=0.6–3.2) were also more likely to be associated with HPV-positive tumours, but none of these associations were statistically significant.

### Human papillomavirus and 11q13 amplification status according to tumour site

**Figure 1:**
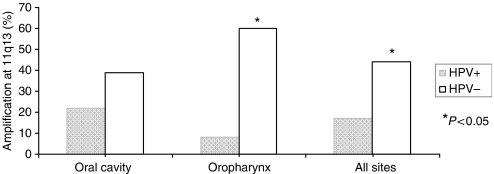
Frequency of 11q13 amp according to HPV status and tumour site.

Amplification status at chromosomal band 11q13 was available for 119 tumours from our study population. Approximately 37% (44 out of 119) were amplified at 11q13. Figure 1 shows the frequency of 11q13 amp stratified by tumour site and HPV status. Overall, we detected a lower prevalence of 11q13 amp in the HPV-positive tumours than in the HPV-negative group (17 *vs* 44%, respectively, Fisher's exact *P*=0.009). In the oral cavity, 22% of the HPV-positive tumours were amplified at 11q13, whereas 39% of the HPV-negative tumours had 11q13 amp (Fisher's exact *P*=0.270). In the oropharynx, a significantly smaller proportion of HPV-positive tumours had 11q13 amp when compared with HPV-negative tumours (8% HPV-positive *vs* 60% HPV-negative, Fisher's exact *P*=0.008).

### *TP53* mutation status, p16 expression and 11q13 amplification status in human papillomavirus-positive tumours

In a subset of tumours (*n*=69) for which data on all three molecular markers were available (i.e. *TP53* mutation status, p16 expression and 11q13 amp status), their associations with HPV status was assessed (Table 3). *TP53* mutations were less likely to occur in the HPV-positive HNSCC (adjusted OR=0.4, 95% CI=0.0–3.1). As expected, HPV-positive tumours were more likely to express p16 (adjusted OR=3.0, 95% CI=0.9–9.7). Sixty percent (nine out of 15) of the HPV-positive tumours exhibited high expression of p16 whereas 33% (18 out of 54) of HPV-negative tumours expressed high levels of p16.

We classified the subpopulation according to combined p16 expression, *TP53* mutation status and 11q13 amp status. Class II included tumours that carried wild-type *TP53*, expressed p16 and was not amplified at 11q13, class III included tumours that also carried wild-type *TP53* and expressed p16 but were amplified at chromosomal band 11q13 and class I tumours were those with all other combinations of *TP53* mutation status, p16 expression and 11q13 amp status. Human papillomavirus-positive tumours were more likely not to be amplified at 11q13 and simultaneously overexpress p16 and contain wild-type *TP53* (class II: adjusted OR=6.5, 95% CI=1.8–23.9). The tumours that contained wild-type *TP53* overexpressed p16, but those with 11q13 amp were less likely to be HPV-positive than those without 11q13 amp and were no different from all other tumours (class III: OR=0.9, 95% CI=0.1–8.9).

## DISCUSSION

This retrospective study of 125 patients with first primary head and neck tumours evaluated the association between tumour HPV status and patient demographics, clinical risk factors and amplification status at chromosomal band 11q13. We further evaluated the relationship between genetic alterations: *TP53* mutation status, 11q13 amp and p16 protein expression in a subpopulation of 69 unselected tumours. Our analysis shows that along with wild-type *TP53* and p16 overexpression, HPV-positive tumours were less likely to carry gene amplification of chromosomal band 11q13, which further defines the characteristic of HPV-positive head and neck tumours and suggests a distinct molecular pathway for HNSCC development.

The relationship between LOH at *TP53*, 11q13 amp and HPV has been shown in one study including only 37 HNSCC (Rodrigo *et al*, 2002). Loss of heterozygosity at *TP53*, 11q13 amp and HPV status were reported on only 11 (30%) of their samples and p16 overexpression was not evaluated. The study reported a lower frequency of 11q13 amp in HPV-positive tumours (HPV+ with 11q13 amp=2 out of 4 (50%); HPV− with 11q13 amp=5 out of 7 (71%) but their HPV-positive tumours were more likely to have LOH at *TP53* (three out of four (75%)). Earlier studies utilising immunohistochemistry have shown that the overexpression of p53 and p16 and reduced expression of cyclin D1 were associated with HPV-positive tumours but 11q13 amp was not evaluated and these studies only included tumours arising in the tonsils (Andl *et al*, 1998; Li *et al*, 2004). One other study evaluated the combined relationship between the three molecular markers and HPV status using immunohistochemistry (Wilczynski *et al*, 1998), but similar to the earlier mentioned studies, their analysis was limited to tumours that arose in the tonsils. In addition, the study included tumours that were first primaries, recurrences, lymph node metastases and lung metastases. Despite the differences in sample size and tumour site included in these analyses, the results from these three studies are in agreement with our findings. In our study, the combined characteristics of *TP53* mutation status, p16 protein expression (a protein which is overexpressed as a consequence of HPV E7 expression) and 11q13 amp (a genetic alteration frequently observed in head and neck tumours) were evaluated to further characterise the role of HPV in head and neck tumourigenesis. Tumours that were HPV-positive were more likely to carry all three markers (wild-type *TP53*, express p16 and no amplification at 11q13). We included in our analyses tumours that were first primaries from various sites within the oral cavity and pharynx and demonstrate that HPV-positive tumours with this distinct molecular phenotype can arise not only in the oropharynx (50%, four out of eight) but also in the oral cavity (50%, four out of eight).

Our results shed further light on the multiple genetic alterations that are commonly found in of head and neck tumours (Gollin, 2001). A genetic progression model for HNSCC has been proposed by Califano *et al* (Califano *et al*, 1996, 2000) and describes a series of ordered genetic changes which may occur during tumour development. It is thought that, for the most part, early genetic alterations include loss of chromosomal bands 9p21, 3p, 17p13 and amplification of 11q13. The candidate genes involved in these genetic alterations are thought to be *CDKN2A*/p16, the tumour suppressor gene *FHIT*, *TP53* and the *CCND1* oncogene, respectively. Examination of hyperplastic, dysplastic, carcinoma *in situ* and invasive carcinoma lesions revealed increasing LOH which corresponded with histopathological progression (Califano *et al*, 1996). Loss of heterozygosity at 9p21 was the most frequent alteration in benign hyperplastic lesions, followed by LOH at 3p21 and 17p13. In addition, dysplastic lesions revealed an increased incidence of allelic imbalance at 11q13 (benign hyperplasia: (6%), dysplasia: (29%), carcinoma *in situ*: 40%, invasive carcinoma (61%)). Our results show that HPV-positive tumours are less likely to be amplified at 11q13 and at the same time p16 is overexpressed, providing indirect evidence that the infection may have occurred at an earlier time point in the carcinogenic process.

The loss of functional *TP53* by mutation seems not to be necessary in HPV-positive HNSCC, as p53 protein loss results from increased degradation in these tumours (Wiest *et al*, 2002; Hafkamp *et al*, 2003; Braakhuis *et al*, 2004). The HPV E7 oncoprotein binds and degrades the RB1 tumour suppressor protein (Boyer *et al*, 1996), which in turn causes the release of E2F (a transcriptional regulator of cell proliferation genes) from pRb/E2F complexes, permitting E2F to transactivate S-phase-related genes. The functional inactivation of pRb by E7 leads to overexpression of the cyclin-dependent kinase inhibitor p16 (Khleif *et al*, 1996). The detection of p16 expression therefore is considered to be a surrogate marker for HPV infection, this observation was also confirmed in our studies.

Amplification of chromosomal band 11q13 has been reported in a number of carcinomas (head and neck, breast, lung, pancreatic, prostate, ovarian, bladder and so on.) (Schwab, 1998). Approximately 45% of HNSCC have amplification of 11q13 (Schuuring, 1995; Lese *et al*, 1995). The amplification status of chromosomal band 11q13 was determined in our study, using a sensitive methodology, FISH with probes for *FGF3/INT2, FGF4/HST1* or *CCND1* (Lese *et al*, 1995; Shuster *et al*, 2000). A significantly smaller proportion of patients with 11q13 amp were observed in the HPV-positive group (13%, two out of 15) compared to the HPV-negative group (54%, 26 out of 48). One consequence of 11q13 amp is *CCND1* overexpression, which is thought to play a direct role in this disease (Callender *et al*, 1994). Other proto-oncogenes which map to the 11q13 core region are amplified and overexpressed (Huang *et al*, 2002), but their role in head and neck tumour development has not yet been fully delineated. The *CCND1* gene product, cyclin D1 associates with cyclin-dependent kinase 4 and 6, and this complex promotes RB1 phosphorylation and like the HPV E7 oncoprotein, their interaction leads to the dissociation of pRb from the transcription factor E2F. This results in transition of the cell from G_1_ into S-phase. One possible explanation for the lack of 11q13 amp in HPV-positive tumours may be that amplification of chromosomal band 11q13 might be unnecessary, as the resulting interaction of the HPV E7 oncoprotein with pRb might allow the cells to be less dependent on *CCND1* for cell cycle progression. We therefore believe that the low frequency of 11q13 amp observed in our HPV-positive head and neck tumours may be explained by a distinct mechanism for HPV carcinogenesis.

Several studies report that patients with HPV-positive head and neck tumours have an improved prognosis (Ritchie *et al*, 2003; Schlecht, 2005), whereas amplification of 11q13 has been associated with a more rapid and frequent recurrence of disease (Fujii *et al*, 2001) and poorer survival (Akervall *et al*, 1997; Rodrigo *et al*, 2000; Namazie *et al*, 2002). Weinberger *et al* (2006) has shown that patients with HPV-positive tumours which express high levels of p16 protein and low levels of p53 protein present with a favourable prognosis. We have shown in our study that HPV-positive tumours have high levels of p16 expression and wild-type *TP53* and in addition, a low frequency of amplification at 11q13. Further analyses are warranted to determine whether this lack of 11q13 amp also contributes to the improved prognosis observed in patients with HPV-positive HNSCC.

## Figures and Tables

**Table 1:** Patient characteristics (*n*=125)

**Characteristic**	***N***	**Percent (%)**
*Age* (*years*)		
<35	3	2.40
36–45	9	7.20
46–55	18	14.40
56–65	43	34.40
66–75	37	29.60
76–85	13	10.40
>85	2	1.60
		
*Gender*		
Male	80	64.00
Female	45	36.00
		
*Race*		
Caucasian	117	93.60
Other	8	6.40
		
*Vital status*		
Alive	44	35.20
Dead	81	64.80
		
*Tobacco exposure*		
Never	13	10.40
Former	36	28.80
Current	76	60.80
		
*Alcohol duration/years*		
Never	18	14.40
Social	3	2.40
2–10	6	4.80
11–30	33	26.40
31–50	53	42.40
>50	12	9.60
		
*HPV status*		
Negative	95	76.00
HPV16	28	22.40
HPV16 & 11	1	0.80
HPV16, 33 & (52)^a^	1	0.80
		
Family history cancer		
No	62	49.60
Yes	63	50.40
		
*Tumour stage*		
I	21	16.80
II	28	22.40
III	27	21.60
IV	49	39.20
		
*Subsequent tumour(s)*		
No	61	48.80
Yes	64	51.20
Total	125	100.00

Abbreviation: HPV=human papillomavirus.

a Unable to rule out HPV52 owing to cross-reactivity of the HPV33 probe.

**Table 2:** Association of patient/tumour characteristics with HPV status

	**Any HPV subtype**			
**Characteristics**	**No. positive (*n*=30)**	**No. negative (*n*=95)**	**Unadjusted OR (95% CI)**	**Adjusted OR^a^ (95% CI)**
*Age* (*years*)				
<55	12	18	1.0 (referent)	
⩾55	18	77	0.4 (0.1–0.9)	0.3 (0.1–0.8)
				
*Gender*				
Male	23	57	1.0 (referent)	
Female	7	38	0.5 (0.2–1.1)	0.5 (0.2–1.3)
				
*Race*				
Caucasian	29	88	1.0 (referent)	
Other	1	7	0.4 (0.1–3.7)	0.2 (0.02–2.2)
				
*Tobacco exposure*				
Never	4	9	1.7 (0.5–6.1)	1.7 (0.4–6.7)
Former	10	26	1.4 (0.6–3.6)	2.4 (0.8–6.6)
Current	16	60	1.0 (referent)	
				
*Alcohol exposure*				
Never	4	14	1.0 (referent)	
Ever	26	81	1.1 (0.3–3.7)	0.9 (0.3–3.2)
				
*Alcohol duration*				
Never user	4	14	1.0 (referent)	
Short-term user	3	6	1.8 (0.3–10.3)	1.3 (0.2–8.4)
Long-term user	23	75	1.1 (0.3–3.6)	0.9 (0.3–3.2)
				
*Combined tobacco and alcohol exposure*				
Ever/Ever	23	76	1.0 (referent)	
Never/Ever	3	5	2.0 (0.4–8.9)	1.8 (0.4–8.6)
Ever/Never	3	10	1.0 (0.3–3.9)	1.3 (0.3–5.6)
Never/Never	1	4	0.8 (0.1–7.8)	0.8 (0.1–8.3)
				
*Family cancer history*				
No	13	49	1.0 (referent)	
Yes	17	46	1.4 (0.6–3.2)	1.3 (0.6–3.2)
				
*Tumour site*				
Oral cavity	18	73	1.0 (referent)	
Oropharynx	12	22	2.2 (0.9–5.3)	2.4 (1.0–5.8)
				
*11q13 amplification status*				
Not amplified	25	50	1.0 (referent)	
Amplified	5	39	0.3 (0.1–0.7)	0.2 (0.1–0.6)
Not performed	0	6	—	
				
*Tumour grade*				
Well	8	24	1.0 (referent)	
Moderate	17	52	1.0 (0.4–2.6)	1.0 (0.3–2.8)
Poor	3	11	0.8 (0.2–3.7)	0.7 (0.1–3.2)
Unknown	2	8	—	—
				
*Tumour stage*				
I & II	10	39	1.0 (referent)	
III & IV	20	56	1.4 (0.6–3.3)	1.4 (0.6–3.5)
				
*Recurrent, metastatic or new primary tumours*				
No	21	41	1.0 (referent)	
Yes	9	54	0.4 (0.2–0.9)	0.4 (0.2–1.0)

Abbreviations: CI, confidence interval; HPV, human papillomavirus; OR, odds ratio.

a Adjusted for age, site and stage, where appropriate.

**Table 3:** Association of molecular markers with HPV status

	**Any HPV**		
**Molecular characteristics**	**No. positive (*n*=15)**	**No. negative (*n*=54)**	**Adjusted^a^ OR (95% CI)**
*11q13 amplification status*			
Not amplified	13	26	1.0 (referent)
Amplified	2	28	0.1 (0.0–0.7)
			
*TP53 mutation status*			
Wild-type	14	45	1.0 (referent)
Mutant	1	9	0.4 (0.0–3.1)
			
*CDKN2A/p16 expression*			
Negative	6	36	1.0 (referent)
Positive	9	18	3.0 (0.9–9.7)
			
*Three molecular*^b^ *markers combined*			
Class I	6	39	1.0 (referent)
Class II	8	8	6.5 (1.8–23.9)
Class III	1	7	0.9 (0.1–8.9)

Abbreviations: CI, confidence interval; HPV, human papillomavirus; OR, odds ratio.

a Adjusted for age, site and stage, where appropriate.

b Three molecular markers combined were classified as: class II=*TP53*wt, p16-positive and no 11q13 amplification.

Class III=*TP53*wt, p16-positive and 11q13 amplification.

Class I=all other possible combination of *TP53* mutation status, p16 expression and 11q13 amplification status.
